# Suicide and self-harm content on Instagram: A systematic scoping review

**DOI:** 10.1371/journal.pone.0238603

**Published:** 2020-09-02

**Authors:** Jacobo Picardo, Sarah K. McKenzie, Sunny Collings, Gabrielle Jenkin

**Affiliations:** 1 Department of Psychological Medicine, Suicide and Mental Health Research Group, University of Otago Wellington, Wellington, New Zealand; 2 Victoria University Wellington, Wellington, New Zealand; University of Milan, ITALY

## Abstract

Given concerns about suicide or self-harm content on Instagram, we conducted a systematic scoping review of peer-reviewed English language primary studies published between 2010–2019. Only ten studies had been published. Looking into purposive samples of Instagram posts tagged with self-harm related hashtags, studies report finding self-harm or suicide content in between 9–66% of their studied posts. Studies assessing Instagram’s efforts to tackle such content found they had not been very effective. Despite heterogeneity in study aims, use of terminology, samples, methods of analysis, and study outcomes, we aggregated and distinguished ‘content studies’ and ‘user studies’. Most studies showed concern for self-harm risk, but only one examined the relationship between self-harm posts and actual self-harm behaviours offline. It found such content had negative emotional effects on some users and reported preliminary evidence of potential harmful effects in relation to self-harm related behaviours offline, although causal effects cannot be claimed. At the same time, some benefits for those who engage with self-harm content online have been suggested. More research directly interviewing Instagram users to understand this phenomenon from their perspective is required. Finally, some ethical issues are discussed.

## Introduction

A large body of research has linked media portrayal of suicide to spikes in suicide rates [1,2]. This association may not be causal; however, there are strong concerns that extensive coverage of suicide reported in a sensationalised or glamourised way, especially celebrities suicides, or giving explicit details of self-harming methods is associated with increase in suicidal behaviours among vulnerable people–a contagion or ‘Werther’ effect [1,2]. Previous research has also warned about possible contagion effects related to suicide stories shared on social media sites like Twitter, especially if the suicide stories resulted in significant audience engagement [3]. Conversely, media stories emphasising recovery and capability to get over crises and suicidal behaviours can have a positive influence on vulnerable people–the Papageno effect–reducing their chances of engaging in such behaviours [2,4].

Non-suicidal self-injury [NSSI], defined as the ‘direct and deliberate destruction of body tissue in the absence of intent to die’ [5], is susceptible to social contagion effects in a similar manner, especially among young people [6]. Studies have related exposure to NSSI on traditional and online media with its onset and maintenance [6]. Suicidal and non-suicidal self-injury are clinically two different behaviours driven by different factors and motivations [5], yet they share some conceptual overlap–they both refer to forms of self-harm [7,8]. For the purpose of this research, we will use the term self-harm in a broad sense, to refer to thoughts and behaviours related to intentional self-injury (e.g. cutting oneself on purpose) without distinguishing between suicidal or non-suicidal intention [8].

Concerns have been raised about the proliferation of easy to access and largely unmonitored self-harm related content online, and whether it triggers or increases self-harming and suicidal behaviours offline [9,10]. Yet we have little understanding of how self-harm content online influences actual self-harm behaviours in vulnerable individuals [8,11].

Previous reviews suggest engaging with suicide or self-harm content online has both positive and negative effects [12–17]. Positive effects include reducing feelings of isolation, providing an online supportive community, distress relief, alternative coping methods and self-harming reduction tips. Negative effects include triggering self-harming behaviours, their perpetuation via normalisation and validation, sharing of self-harm methods, and tips for concealment. To date, reviews have focused on the effects of social media and the internet on suicide and NSSI [12,14–17] or self-harm in general regardless of intention [13], with half of reviews focused on young people [12,13,16]. Research in this field has been conducted using different methodologies, with primary research drawing on quantitative, qualitative and mixed methods with diverse research designs [16]. Diane [12] suggested a possible interaction effect between research design and findings, with qualitative and mixed methods studies showing a more positive impact of the internet on self-harm behaviours, compared to the more negative impact reported in quantitative studies.

These reviews [12–17] compiled and reviewed data from sites like Facebook, Twitter, YouTube, and other suicide-related internet forums. None of these reviews included any studies about self-harm or suicide on Instagram. With over one billion active monthly users [18], Instagram is one of the most popular social media platforms among young people [19,20], and a common platform for posting self-harm content [11,21].

Although text-only posts are allowed since 2018 [22], Instagram was originally designed to share pictures [23] and short-videos, standing out amongst other social media platforms because of its visual nature, ‘*sign up to see photos and videos from your friends’* [24]. Instagram content can be tagged using key terms known as ‘hashtags’ (e.g. *#selfharm*). Hashtags allow users to search for and find content of interest and ‘connect’ with others with similar interests [25].

Instagram came under public pressure after several high profile cases of youth suicide were found to have shared and engaged with self-harm content on Instagram [26–28]. Instagram has acknowledged the issue and promised an increased focus on minimising harm from their platform [29]. Meanwhile evidence of the relationship between Instagram content and increased risk of self-harm behaviours is unclear and fragmented [21], a scoping review of the current evidence is needed. Scoping reviews allow for “reconnaissance” of an emerging field of research inquiry [30]. They bring together information from primary research and present it in a cohesive way, clarifying concepts and methods, identifying gaps, and informing next steps [31]. We aim to synthesise how self-harm or suicide content on Instagram has been studied, what we know about it, and identify gaps in the literature to inform future research in the field.

## Methodology

Following PRISMA (S1 File) and Joanna Briggs Institute guidelines for systematic scoping reviews [31,32] we: a) specified a research question; b) elaborated a review protocol (see S2 File); c) identified and selected relevant studies; c) extracted the main data out of the selected studies (data charting); d) collated, summarised, and reported the main findings; e) summarised the strengths and limitations of the body of literature, and reviewed its quality. Findings are summarised narratively and on a table.

### Research questions

What research has been done on the topic of self-harm or suicide on Instagram, how has it been done, and what are its key findings?

### Retrieving relevant studies

An electronic literature search was conducted (on 11/04/2019 and 1/11/2019) for all English language peer-reviewed articles (to learn from evidence up to highest scientific standards and facilitate replication), indexed in Scopus, Web of Science, Medline, EBSCOhost, PsycINFO, EMBASE and ProQuest Central, from 2010 (date when Instagram was launched) onwards, with databases search alert functions providing ongoing updates up to 5/01/2020.

The following search terms were applied to titles, abstracts and keywords to maximise sensitivity: (instagram* OR “insta gram*”) AND (suicid* OR “self harm*” OR selfharm* OR “self injur*” OR selfinjur* OR “self mutil*” OR selfmutil* OR “auto mutil*” OR automutil* OR cut* OR distress* OR disorder* OR anxi* OR depress* OR “psycholog* stress*” OR “psycholog* pain*”). Search terms related to mental health issues like depression, anxiety or psychological pain were also included due to their association with suicide and self-harm. Search strategies and phrasing were database-specific due to indexing differences. Library staff were consulted to retrieve optimum results.

#### Inclusion and exclusion criteria

Studies had to be published in peer-reviewed journals, and explicitly examine suicide, self-harm or non-suicidal self-injury, and Instagram. We excluded non-peer reviewed reports, grey literature, conference papers, theses, books and book chapters, and studies examining social media in general but not explicitly Instagram. No study was excluded because of its quality appraisal.

### Quality appraisal

We did not dismiss any paper on the basis of its quality. The quality assessment of the available studies was performed as a proxy for the state and level of quality of the research field as a whole. For that purpose we used a quality appraisal checklist based on the Critical Appraisal Skills Programme [CASP] [33]. Based on the information reported on the articles, for each study we assessed (*Yes*, *No or Can’t tell)*: whether it included a clear and relevant statement of aims; whether it reported its methodology, research design, sample and data collection adequately to address its research aims; whether it reported rigorous data analysis, clear findings, and acknowledged its limitations; whether relevant ethical issues had been taken into account; and finally we also assessed whether the research was valuable and contributed to the body of knowledge (See S2 File).

JP assessed the quality of all ten reviewed papers, and GJ and SM independently checked half of them each. Afterwards, discrepancies were discussed and resolved as a team.

### Data charting

A data extraction sheet (see S2 File) developed by the authors was used to chart study: a) Identification and introductory information (i.e. authors, date and journal of publication, main author affiliation, study aim, and online social media platforms studied); b) Methodological information (i.e. study design, unit of analysis, data collection strategy and sample characteristics, coded/assessed variables, data analysis performed); c) Main reported findings and conclusions; d) Limitations; e) Overall quality score.

## Results

**Fig 1:**
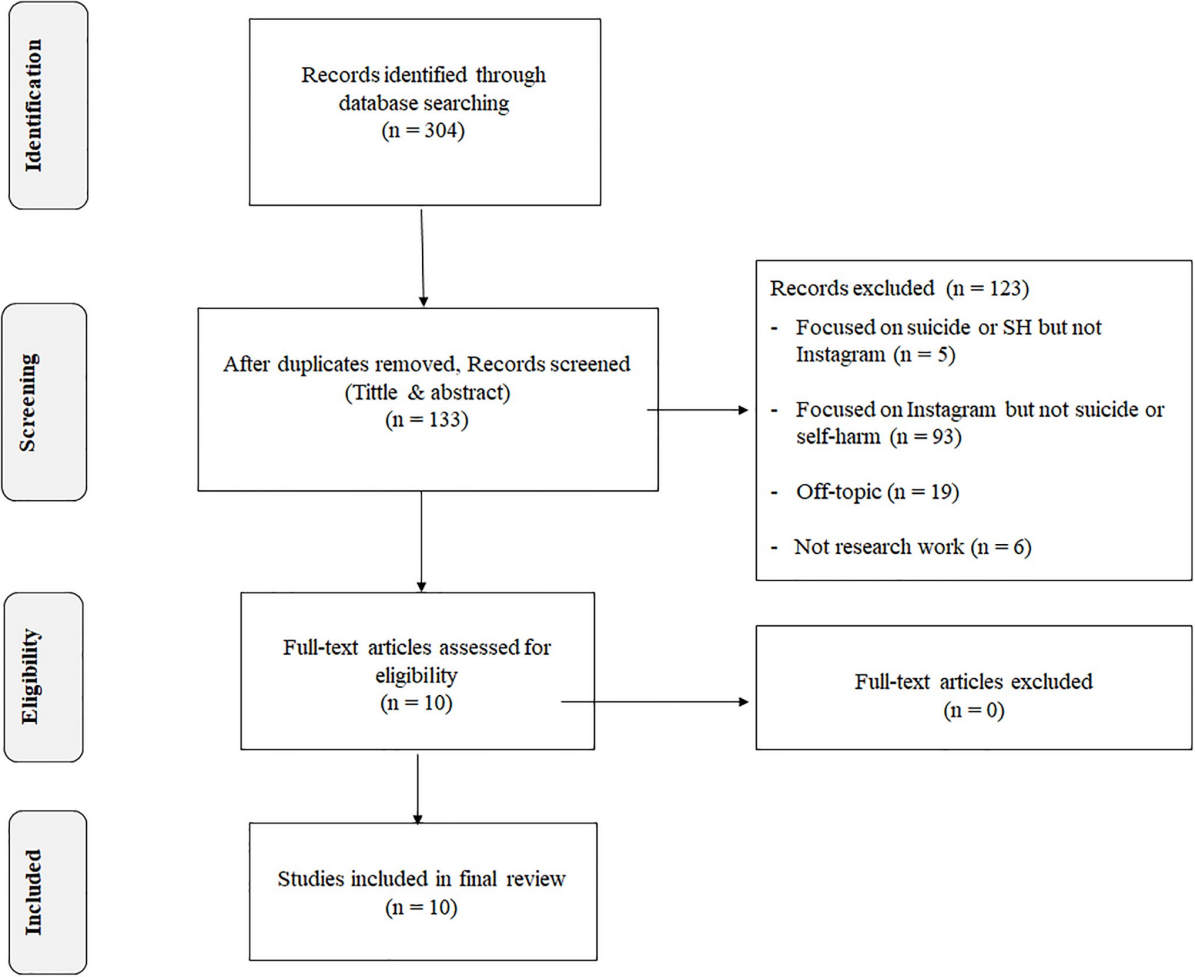
Articles selection. PRISMA flow diagram of articles selection process [31].

The search yielded 304 articles, of which 133 remained after removing duplicates. The first author (JP) completed the initial search and carried out the first stage of the screening process for relevance, based on title and abstract. After applying the inclusion and exclusion criteria 10 articles remained [34–43]. Authors (JP, GJ, SM) independently reviewed the full articles and examined their reference lists. No further relevant articles were identified (Fig 1).

### Studies characteristics and quality

The key characteristics and overall quality score of the reviewed studies are given in Table 1. All studies were published between 2016–2019 at an increasing rate every year. Based on first author, articles originated from US/UK (n = 5), Germany or Austria (n = 4), and Belgium (n = 1). Eight studies focused on Instagram only [34–41], while two examined self-harm across Instagram, Twitter and Tumblr [42,43].

**Table 1:** Key characteristics of included articles.

Reference, year, country	Study Type	Data Collection	Sample	Analysis	Aims, Key Findings (Quality criteria met)
**Studies including qualitative description of Instagram post**					
Moreno [40], 2016 USA	Qualitative and quantitative descriptive study	Extracts Instagram content using the hashtag *#selfharmmm* for 12 days	N = 225 posts	Structured evaluation approach developed by the authors, using content analysis and triangulation.	**Aims**: To investigate ambiguous non-suicidal self-injury (NSSI) related terms on Instagram including evaluation of meaning and consistency. To assess the precision of Instagram’s warning labels. **Findings**: 10 ambiguous NSSI hashtags were identified. A popular image called *#MySecretFamily* was identified that described the broader community of NSSI and mental illness. Only one-third of all relevant hashtags generated Instagram’s content advisory warnings. Key contribution: details a systematic method to ensure rigorous, valid coding of online content. (10/10)
Shanahan [43], 2019 UK	Qualitative thematic study	Extracted the most recent 200 images tagged as *#self-harm* from three social media platforms on one day. (Inclusion of broad SH content, not just limited to NSSI pictures).	N = 602 images	Visual and thematic content analysis of images.	**Aims**: To explore the nature and content of images tagged as #self-harm across Twitter, Instagram and Tumblr. **Findings**: Over half the images tagged as self-harm had no explicit presentation of self-harm. None portrayed images of graphic or shocking self-injury. Four themes were found across the images: communicating distress, addiction and recovery, gender and the female body, identity and belonging. (10/10)
**Quantitative descriptive studies of Instagram posts**					
Miguel [42], 2017 USA	Quantitative descriptive study	Extracting the first 10 posts from Instagram, Tumblr and Twitter using the hashtag *#cutting* every day for 6 months.	N = 770 posts Insta(n = 359) Tumb(n = 333) Twit(n = 78)	Content analysis. Posts coded using a pre-defined list of study codes and definitions.	**Aims**: To analyse and compare NSSI content tagged as #cutting across Instagram, Tumblr and Twitter. **Findings**: 60% of all sampled posts depicted graphic self-harm content (Instagram: 66%), 10% discouraged self-injury (Instagram: 8%) and <1% included formal recovery resources. Instagram posts displayed the greatest proportions of graphic content and negative self-evaluations, followed by Tumblr then Twitter. (10/10)
Brown [37], 2018 Germany	Quantitative descriptive study	Extracted pictures tagged with different German NSSI hashtags from Instagram accounts for 4 weeks.	N = 32,182 images depicting wounds plus 6,651 associated comments.	Quantitative content analysis using descriptive statistics, frequencies and correlations	**Aims**: To investigate Instagram NSSI-pictures and comments. Explored associations between pictures characteristics, comments, and posting trends. **Findings**: 93% of pictures depicted cuts, mostly mild/moderate wounds, while very severe wounds were rare. Pictures with increasing wound grades and those showing multiple types of wounds received more comments. Most comments were neutral or empathetic, few were hostile. (10/10)
Carlyle [39], 2018 USA	Quantitative descriptive study	Extracted randomly selected suicide-themed Instagram posts using *#suicide* and *#suicidal* hashtags for 4 months using an online mining tool.	N = 500 posts	Quantitative content analysis using descriptive statistics.	**Aims**: To examine both visual & textual components of suicide-themed Instagram posts. **Key findings**: Self-harm content was present in the majority of Instagram posts. Posts mentioning suicidal ideation elicited higher engagement from users than posts that did not. Health voices were absent from the online discussions. (9/10)
Scherr [36], 2019 Belgium	Quantitative descriptive study	Extracted all pictures posted on Instagram within a 48-hour period using English and German suicide related hashtags.	N = 13,132 images	Computational methods using artificial intelligence [AI] image-recognition algorithms developed by the authors.	**Aims**: To develop an AI image-recognition algorithm able to automatically identify NSSI images online and use it to identify and quantify NSSI images on Instagram. **Findings**: The image-recognition algorithm successfully identified the proportion of NSSI images tagged as *#suicide* = 40% (n = 7,910), *#cutting* = 30% (n = 4,219), *#selbstmord* = 40% (n = 173) and *#ritzen* = 42% (n = 830). (10/10).
Arendt [34], 2019 Germany	Quantitative descriptive study	Extracted the most recent Instagram posts tagged with *#selbstmord* (‘suicide’) on one day.	N = 250 posts	Quantitative content analysis using descriptive statistics.	**Aims**: To examine Instagram posts tagged as *#selbstmord*, (‘suicide’ in German). **Findings**: 46% of sampled posts included explicit references to suicide. Cutting was the most prominent method. Sadness appeared as the most described emotion, followed by self-hate and loneliness. Video content made up 26% of sampled posts, many of which presented very fast/subliminal suicide content. (10/10).
**Quantitative survey studies of Instagram users**					
Record [41], 2019 USA	Quantitative survey	Online survey of college students with Instagram accounts	N = 471 undergraduate Instagram users (60% white and 77% female)	Cross-sectional analysis.	**Aims**: To evaluate users’ awareness of and intention to use the Instagram self-harm reporting tool, according to the theoretical principles of diffusion of innovation and planned behaviour (TPB). **Findings: <**20% of users knew about the self-harm reporting tool. The frequency of Instagram use was unrelated to the intention to use it. Instead, users’ attitudes, subjective norms and behavioural control over reporting SH on Instagram positively related to their intention to use it. (10/10).
Arendt [35], 2019 Austria	Quantitative panel survey	Online survey of young adults	N = 729 participants (with data at waves 1 and 2) 18–29 years	Cross-sectional and longitudinal analysis.	**Aims**: To investigate the relationship between exposure to self-harm content on Instagram and suicidal behaviours outcomes. **Findings**: 43% of participants had seen SH-content on Instagram (most often found accidentally). Exposure to self-harm on Instagram was related to suicidal ideation, SH and emotional disturbance. (10/10).
**Semi-structured interview studies of Instagram users**					
Brown [38], 2019 Germany	Quantitative and qualitative descriptive study	Semi-structured online interview with Instagram users, extraction of users’ posts captions and overall Instagram activity.	N = 52 Instagram users (who had posted self-harm pictures and reported lifetime history of suicidal ideation).	Quantitative linguistic analysis of participants’ discourse and Instagram captions. Plus, thematic analysis of participants’ accounts of audience reactions to SH/Suicide posts.	**Aims**: To investigate users experience with suicidal expressions on Instagram. To analyse users’ language use during interviews and on Instagram postings. **Findings**: 80% of participants report having seen acute suicidal expressions on Instagram, and 25% of them had posted their suicidal thoughts or plans. Users’ activity and language used on Instagram did not distinguished participants with current suicidal ideation. Acute suicidality was only associated to participants increased use of negative emotions expressions during interviews. (10/10)

^a^ ‘Post’ = main image/text or video, plus captions or hashtags (visual and textual components as a whole).

Seven of the ten studies retrieved and described self-harm or suicide related content on Instagram [34,36,37,39,40,42,43]. One study assessed Instagram users’ awareness and use of the Instagram reporting tool for self-harm content [41]. Arendt [35] measured the relationship between exposure to self-harm content and users’ self-harm behaviours and suicidality offline, and Brown [38] explored whether online users’ acute suicidality could be predicted from their Instagram activity and language used in their posts captions.

All reviewed studies were high quality articles (see S1 Table for the detailed quality appraisal scores). Nine articles scored ten out of ten in overall quality [34–38,40–43], and Carlyle [39] scored nine out of ten. All reported adequate aims, good methodology, research design, sample, rigorous analysis, and clear and adequate findings, bringing valuable research contribution to the field. All openly acknowledged some limitations, typically limits to extrapolate beyond their purposive samples [34–38,40–43] or lack of Instagram users’ information [35,42,43]. In terms of ethical considerations, most studies report having IRB ethical approval [34,35,37,38,40–43] or having followed ethical guidelines approved by the ethics committee of the Association of Internet Researchers (https://aoir.org/ethics/) [36]. Carlyle [39] reported none of those. Instead they claimed that ‘because the study did not involve human subjects, it did not require ethical reviews.’

### Studies self-harm terminology

Terminology across studies was heterogeneous, using the various terms of ‘self-harm,’ ‘deliberate self-harm’ or ‘deliberate self-injury.’ Some used these terms more broadly, to capture suicidal or non-suicidal self-injury content [35,41–43], or narrowly to capture NSSI only [36]. In particular, Scherr [36] and Brown [37] studied NSSI images on Instagram. Others claimed to study suicide content on Instagram [34,39]. However, none of these Instagram content studies [34,36,37,39,42,43] undertook any contact or follow-up with the person posting such content; therefore, their suicidal/non-suicidal meaning or intention cannot be confirmed. Only Moreno [40] added data triangulation (on top of commonly used inter-coders agreement) to validate the meaning of self-harm related hashtags.

### Studies methodological differences

Samples size and sample frames varied widely (see Table 1). Seven out of ten studies used samples of publicly available Instagram content [34,36,37,39,40,42,43]. Three surveyed or interviewed Instagram users [35,38,41].

#### Instagram user studies

Three studies asked online users about self-harm or suicidality on Instagram [35,38,41]. Record [41] used an online survey of college students (n = 417) to report their Instagram use, their awareness of Instagram’s tool for reporting self-harm content, and factors related to their intention to use it. Arendt [35] designed a two wave follow-up study, using an online survey to ask 18–29 year old Instagram users (n = 1000 at Time 1 and n = 729 at Time 2) about their exposure to self-harm content on Instagram, and other self-harm related outcomes offline (ideation, risk, hopelessness, emotional disturbance and behaviours), controlling for exposure from other sources. Both studies were based on convenient samples of self-selected participants recruited through wider online platforms [35,41]. Brown [38] used Instagram messenger to interview 52 young people (average age 16), who had shared and tagged content on Instagram as self-harm or suicide-related (purposive sample). They analysed users’ language used during interviews and in their Instagram publications to try and identify those with acute suicidal ideation [38].

#### Instagram content studies

All other studies [34,36,37,39,40,42,43] focused on Instagram’s self-harm or suicidal content, studying pictures, text images, captions, hashtags or comments. Authors explored Instagram pictures and posts tagged with hashtags like: *#suicide* or *#suicidal* [39], *#self-harm* [43], *#selfharmmm* [40], *#cutting* [42], and analogous hashtags in German, like *#selbstmord* [‘suicide’] [34,37] or *#ritzen* [‘cutting’] [37]. Moreno [40], followed a systematic approach to identify ambiguous hashtags of self-harm related content on Instagram. Despite the concealing nature of such hashtags, they confirmed their self-harm meaning using data triangulation, checking whether hashtags were used consistently across multiple platforms to tag self-harm related content.

These studies relied on human coders to identify self-harm related hashtags and content on Instagram [34,37,39,40,42,43]. This approach has important limitations: self-harm related hashtags change constantly and are easily outdated; inter-reliability issues where more than one coder is used; limits to the quantity of content that can be coded by humans at any one time; and the need for a time lag between the content being identified, retrieved and coded as ‘self-harm/suicide related’ [36]. Scherr [36] looked to resolved some of these issues by developing and testing the first artificial intelligence [AI] based image-recognition algorithm to automatically identify NSSI pictures of cuts on Instagram. They first trained the AI using 600 pairs of images depicting a) NSSI related cuts (manually identified from Instagram), against b) not-NSSI pictures. The accuracy of the NSSI-identifying algorithm was tested against a different set of cutting/not-cutting pictures. Once the algorithm reached a good classifying performance, the authors automatically downloaded all pictures (N = 13,132) from Instagram tagged with German and English self-harm/suicide related hashtags (*#cutting/#ritzen*, *#suicide/#selbstmord*) during 48h, and used the automatic AI to quantify the amount of NSSI Vs No-NSSI content attached to each hashtag, and compare the chances of encountering NSSI content when using the different English or German hashtags [36].

#### Studies methods of analysis

Most studies used thematic or content analysis to explore and describe self-harm or suicide content on Instagram, reporting descriptive techniques, frequencies, and statistics [34,37,39,42,43]. Some studies partially quantified the amount of self-harm related content on Instagram [36,40]. Moreno [40] details a systematic approach followed to ensure rigorous and valid coding process of online content, she used it to uncover the self-harm related meaning of different ambiguous hashtags used on Instagram, reported the raw number of posts tagged with such self-harm-hashtags on the platform (potentially self-harm content), and measured Instagram’s capability to identify such content (before 2016). Brown [37] used content analysis to describe cutting-NSSI pictures on Instagram, audience response, and record time trends of NSSI-postings.

Instagram posts are qualitative in nature, yet content analysis relies on counting and comparing frequencies of coded characteristics of interest [44], reporting quantitative analysis and statistics (frequencies, chi-square, or Mann-Whitney U tests) [37,39,42]. Carlyle [39] stated using “quantitative content analysis” to study suicide content on Instagram.

Brown [38] used quantitative linguistic inquiry and word count to analyse data from online-interviews with Instagram users, their language used on Instagram posts captions, and quantification of their general Instagram activity. T-test statistics were used to find differences between users with current versus past suicidal ideation. They also reported qualitative thematic analysis of participant account of online audience responses to self-harm or suicidal posts [38].

Survey studies of Instagram users applied regression analysis to estimate what factors would predict user utilisation of Instagram’ self-harm reporting tool [41], and the relationship between exposure to self-harm content on Instagram and deliberate self-harm offline (both cross-sectionally and longitudinally) [35].

### Studies main findings

#### Self-harm or suicide content reported on Instagram

Out of the different samples of Instagram content studied (between 225–32,182 Instagram posts tagged with different self-harm or suicide related hashtags), studies report finding actual self-harm/suicide related content in around 9 to 66% of the examined posts [34,37,39,42,43]. The nature of such content was diverse, with some content explicit and some less so (e.g. pictures of wounds, objects/paraphernalia, selfies, drawings, memes, short videos, text images, references to movies or songs, quotes) [34,37,42,43]. Suicidal intent was reported as present in 19% of studied English language posts tagged as *#suicide* or *#suicidal* (N = 500), with 46% of them being text-based images, against 20% of actual depictions of wounds [39]. 61% of those #suicide/suicidal posts also mentioned ‘self-harm’[39]. Arendt [34] found that, out of their sample (N = 250) of German suicide posts (tagged *#selbstmord* [‘suicide’]), 46% made explicit reference to suicide. Of these, 26% were fast/subliminal-like videos depicting self-harming behaviours [34]. Brown [37] found that 9% of pictures tagged with German NSSI-related hashtags (N = 32,182) were explicit self-harm images. Across Twitter, Tumblr and Instagram, Miguel [42] reported that 60% of their sampled posts (N = 770 tagged as *#cutting*) were pictures of blood, cuts, scars or other injuries, self-injury paraphernalia, and/or active self-injury. Instagram (n = 359) hosted the greatest proportion of visual self-harm content (66%) and the lowest proportion of help or recovery-oriented posts [42].

Overall studies agree that depictions of mild-moderate severity cuts (usually on arms or legs) are the most common explicit self-harm related content found on Instagram [34,37,43]. However, only Brown [37] clearly operationalised severity (i.e. *‘mild’*: *superficial scratches; ‘moderate’*: *deeper cuts or showing blood; ‘severe’*: *very deep*, *gaping cuts or large amount of deeper cuts and blood*). Some authors identified such content as NSSI depictions [37,39,43], although they failed to explain how they distinguished intentionality, and they did not provide any examples of the posts. Shanahan [43] warned that ‘identifying stated purpose and tone [of the images] was difficult as often images were ambiguous.’

Authors agree that self-harm content online represent clear expressions of posters’ distress and struggle, often linked to references to sadness, loneliness, negative feelings, and related mental health problems such as depression and eating disorders [34,39,42,43]. Self-harm on Instagram was often referred to as an addiction, and the sharing of posts as part of the process towards recovery [43].

Studies report self-harm content on Instagram receiving high volume of audience engagement and attention, with visual and more gory posts receiving a greater number of ‘likes’ [37,39]. Other users’ comments most often showed empathic support and care [37–39]. Brown’s [37] hypothesis that social reinforcement was behind users posting such content was not proven. Others suggest that users post self-harm content online as a way to reach out and receive empathy [43].

Suicide and self-harm content on Instagram are usually tagged online using self-harm- specific hashtags like *#selfharmmm* [40]. Using content analysis and data triangulation, Moreno [40] showed that such content is often shared using ambiguous and concealing self-harm related hashtags, to avoid Instagram censorship (e.g. *#selfharmmm or #selfinjuryyy*, *#Blithe/#ehtilB*, *#cat* [meaning cut], *#sue* [for suicidal] or *#mysecretfamily*). Online communities have emerged around these hashtags, allowing users with self-harm or suicide interests to come together online [40]. Moreno [40] found there were great amounts of posts tagged with such hashtags on Instagram, and that Instagram’s ability to flag such content was limited, as hashtags evolved faster than they could be tracked and assessed by Instagram content moderators [36,40].

To address this, Scherr [36] developed and tested an artificial intelligence (AI)-based image-recognition algorithm able to distinguish self-harm content by cutting with an 87% accuracy. Using the algorithm to find self-harm pictures on Instagram, they estimated that users using the German self-harm hashtag (*#ritzen*) were 39% more likely to find explicit self-harm content than using the equivalent English hashtags (*#cut*), whereas other suicide-related hashtags (*#selbstmord or #suicide)* had a similar risk (-0.03%) [36].

#### Level of concern about self-harm posts

Most authors expressed concerns about the danger for contagion that self-harm or suicide content on Instagram may pose to those who engage with it [34,37,39,40,42]. Researchers argued that such content on Instagram does not follow media reporting guidelines to avoid contagion [39]: Instagram exhibits explicit references to self-harm methods, and shows a paucity of help, recovery-oriented, and professional driven content [34,39,42]. Some warn such content may normalise self-harm behaviours as a way to cope, increase the risk of imitation, and be triggering for vulnerable users [37,40]. However, Shanahan [43] claims that we should not be overly anxious about self-harm content shared on Instagram. Shanahan [43] saw such posts as depicting mild, not dangerous content, and more as manifestations of distress and negative emotions rather than sensationalised invitations to self-harm. However, none of these descriptive studies of content were set to test contagion effects. That would require designing prospective studies to measure exposure effects.

Survey data showed that out of a sample of 729 young adults, 43% had at some point been exposed to self-harm content on Instagram (20% of those searched for it intentionally) [35]. Users found such content disturbing, and 33% of them indicated having performed ‘the same (or very similar) self-harming behaviours as a consequence of seeing self-harm content on Instagram’ [35]. Cross-sectional analysis showed that lifetime exposure to self-harm content on Instagram was significantly correlated with self-harm behaviours (r(302) = .40, p < .001), suicidal ideation (r(280) = .27, p < .001), hopelessness (r(302) = .26, p < .001), reasons for living (r(303) = -.17, p = .002), and suicide risk (r(302) = .40, p < .001) [35]. Moreover using longitudinal data, and controlling for initial previous vulnerability (self-harming outcomes at Time 1), exposure to Instagram self-harm content at Time 1 was found to be positively correlated with increases in self-harming behaviours, suicidal ideation and hopelessness; as well as being negatively related with reasons for living at Time 2 [35]. However, exposure to self-harm content did not show any effect on suicide plans [35].

Looking into a more specific sample of 52 Instagram young users, with previous history of suicide ideation, and having shared self-harm content on Instagram, Brown [38] reported that 81% had seen expressions of suicidal thoughts on Instagram, and 25% had expressed their own suicidal thoughts at some point on Instagram. At the time of the interview, 25 users were experiencing current suicidal ideation, and 12 had made a suicide attempt in the last year. However, based on their overall Instagram activity and posts captions (using quantitative linguistic inquiry and word count analysis), it was not possible to distinguish those users with current suicidal ideation from those with just past history of suicidality [38]. The only significant difference between those with acute suicidal thoughts compared to those with past suicidality was how participants talked during direct messenger-based interviews [38], with participants with acute suicidality using significantly more negative-emotional and affective words [38]. The amount of negative-emotional words (e.g. “sad,” “angry”) was the only characteristic able to predict acute suicidality (cut-off = 0.7 for 67% accuracy, 84% sensitivity, and 57% specificity) [38].

#### Countermeasures

There is no consensus around how self-harm content on Instagram should be managed. Record [41] showed that Instagram’s reporting tool for self-harm content had not been very successful, probably because less than 20% of Instagram users surveyed knew about it. Other suggested possibilities to deal with self-harm content on Instagram included hindering access to such content by rendering suicide and self-harm-related hashtags unusable [42], and increasing the presence of help-seeking content [39,42], neither of which have been formally tested.

## Discussion

Previous studies had shown that social media is a common platform for youth to post about self-harm or suicide [13,45]. Instagram is a key social media platform for young people [21], a group of particular interest for suicide and self-harm prevention [8]; however, there has been paucity of published research about suicide or self-harm on Instagram. This review shows that this is changing, and that a small corpus of good quality scientific literature is starting to emerge.

The actual prevalence of self-harm or suicide content on Instagram is unknown. Unveiling this would require access to the full pool of Instagram posts, something only feasible for Instagram itself. Instead, current studies retrieved different purposive samples of Instagram content, publicly shared with self-harm or suicide related hashtags. Studies reported finding self-harm or suicide related content in around 9–66% of their examined posts [34,37,39,42,43]. The different quantity of self-harm or suicide content reported across studies is to be expected, given studies sampling and methodological differences. Some study samples included any Instagram post tagged as *#self-harm*, in a broad way, regardless of its actual content [43], others focused only on pictures of cuts [36,37], or reported how many of their sampled users recalled having seen suicidal content on Instagram [38]. Studies use different search terms, which return different amount of self-harm content (e.g. German NSSI-hashtags showed to retrieve greater proportion of actual NSSI-content than its counterpart in English) [36]. The date when the content was collected also matters, as Instagram made changes to its content policy regarding self-harm [21,29].

Altogether, studies used different approaches to research suicide or self-harm on Instagram. We distinguished between ‘content studies’ and ‘users studies’ depending on their sample and how they collected their data. ‘User studies’ contacted Instagram users directly, using surveys and online semi-structured interviews, to ask them about different aspects in relation to self-harm or suicidal content on Instagram [35,38,41], in some cases also mining their Instagram activity and personally uploaded posts captions [38]. Instagram ‘content studies’ collected publicly posted content tagged with different suicide or self-harm related hashtags on Instagram, without the actual users’ involvement in the process (or even being aware of it). Some examined hashtags, some pictures only, and some pictures and captions together as whole posts, with some including other users response to such content [34,36,37,39,40,42,43]. One study used AI to automatically distinguish pictures of NSSI-cuts from not NSSI-pictures, and used it to quantify and compare the amount of actual NSSI-pictures associated to different self-harm and suicide related hashtags in English and German [36].

Studies varied in their approach to analyse their data. Content studies typically used descriptive content analysis to code and characterise self-harm or suicide content. Studies used different coding protocols, but in general reported the amount of self-harm content present among their sample, with some also including audience responses, content time trends, or quantifying Instagram ability to identify such content [34,36,37,39,40,42]. Only Shanahan [43] offered a thematic description of self-harm content on Instagram and social media. All these content analysis studies relied on inter-coders agreement for reliability. Moreno [40] detailed a method that adds data triangulation, to foster rigour and validity in the coding of online content.

Survey studies carried out regression model-based analysis [35,41] and Brown [38] used quantitative linguistic inquiry to analyse users’ language on Instagram.

A previous review about self-harm and suicide online suggested the possibility of interaction bias between study designs and study outcomes, by which qualitative studies tended to find self-harm content online to be less problematic than quantitative studies [12]. In our review most studies using content analysis showed concerns about the proliferation of suicide or self-harm content on Instagram, whether such content and the community of users around it may normalise and reinforce self-harming behaviours, or even maybe facilitate social contagion among vulnerable users [35,39,34,37,40,42,36]. Only Shanahan [43] thematic study concluded that we should not over-worry about self-harm content online, that it is an avenue for expressing difficult emotions, more than a glamorised incitation to self-harm. However, only one study actually looked into the relationships between engaging with self-harm content on Instagram and self-harm or suicidal correlates offline [35]. It reported cross-sectional and preliminary longitudinal negative effects on self-harm and suicide-related outcomes offline [35]. Still, authors were cautious not to claim causal exposure effects. Whether people are at greater risk of self-harm because they engage with such content online, or rather they end up engaging with it because they were at greater risk for self-harm to begin with (reverse causality) cannot be ruled out based on the current evidence. Other confounders may be driving such relationship [35]. This is a common limitation of survey studies in the broader field of research [2,9]. However, Arendt’s study [35] adds to the voices raising concerns about harmful effects of self-harm content online, including compelling data about potential for copycat effects.

Terminology was another source of heterogeneity. Terms like self-harm, deliberate self-harm, self-injury or non-suicidal self-injury are all used to refer to similar (if not the same) behaviour, and they are often used interchangeably. This reflects the lack of consensus over terminology use more broadly in the field of suicide and self-harm research generally [5,46]. This is relevant because terminology can have methodological implications, influence study findings, and their interpretation. For example using *#selfharm* or *#suicide* to retrieve posts, and assume to study NSSI or suicide on Instagram [34,37,39,40]. Brown [37] coded as NSSI all pictures of wounds tagged with self-harm-related hashtags in German. Yet, Carlyle [39] found that the majority of *#suicide*-tagged posts also mentioned self-harm. Accurately distinguishing between suicidal or not suicidal self-harm behaviours and content is important because they are different things; however, this can be challenging [47]. When examining self-harm content online, unless we know more about the people posting it and their motives, distinguishing between self-harm content that is suicidal or non-suicidal is highly problematic [43]. In future research, one strategy might be to use the term ‘self-harm’ in a broad sense, to refer to behaviours and intentional acts carried out on oneself, knowing that they would cause pain or harm, regardless of suicidal intention, excluding alcohol abuse, smoking and other recreational drug use, and accidental harms to oneself [48].

Studies seem to agree that self-harm content represents users’ experience of distress [34,43]. The nature of such content is diverse, but most often it involves depictions of cuts. Compared to other social media, Instagram was found to host the greatest proportion of visual self-harm content [42]. This is relevant because previous research has suggested that imagery and visual content may have greater impact on users than text, it is more appealing, attracts more attention and may have greater triggering potential [45,49]. We found that self-harm content on Instagram elicits high levels of audience engagement, especially the more graphic and explicit it is [37,39].

Similarly to previous studies about pro-eating disorders content on Instagram [50], our review found that self-harm and suicide content on Instagram is typically shared using self-harm-specific hashtags, around which online communities emerge [40]. Such hashtags are designed to avoid Instagram censorship and this appeared to be effective [40]. Surveillance methods drawing on hashtag and captions to identify self-harming content do not appear to be very effective [40]. Moreover, Instagram’s tool for users to report negative content has not been widely adopted [41]. New technologies such as AI automatic image recognition offer a possible solution [36] and Instagram is looking into their implementation [21].

## Strengths and limitations

This is the first review of primary studies around self-harm and suicide content on Instagram. Our search strategy was limited to English-language publications, but was not limited to any particular age group, despite the usual adolescent age focus of previous internet reviews [12,16]. Our review did not include grey literature as we focused on peer-reviewed publications due to their scientific quality and likely replicability. Our quality appraisal of the current literature provides a good proxy of the overall quality the field as a whole.

## Implications and future research

There is self-harm content on Instagram. Although scarce, most research to date has focused on describing it. Most scholars show concerns about such content [34–37,39,40,42], however, there may also be some benefits for those who engage with it [15,43]. How self-harm content on Instagram relates to users self-harm risk and behaviours offline has been understudied, but researchers are starting to look into it and explore the factors underlying this relationship [35]. More research is needed to expand this line of inquiry [21,35]. Future research should move on beyond mere description of Instagram content.

Brown [38] showed that looking at the online activity and content of those sharing self-harm or suicidal posts on Instagram alone was not enough to accurately identify those at higher risk of suicide. Only by directly chatting with the posters themselves were they able to distinguish those with current versus past suicide ideation [38]. Accurate, reliable information about the users engaging with self-harm content online is needed [35,42,43]. We need more qualitative research, directly approaching online users who engage with such content, to obtain reliable users’ information, and better understand what in their views constitutes self-harm content online, why they engage with it, how it affects them, and relates to them offline. We need to understand this online phenomenon from the users’ perspective. At the same time, research could examine the potential of positive narratives and content, as suggested by the potential of the Papageno effect, to focus on users’ experiences of getting through suicidal crises and self-harming behaviours [4]. As in the broader field of suicide and self-harm research, consensus and correct use of self-harm terminology is needed. Finally, consensus around good ethical practises in this field of research is needed. Some content studies undergo IRB review [34,37,40,42,43], some do not [36,39]. Previous guidelines back IRB exception for observational studies of social media content (under certain conditions) [51]. However, given the sensitive nature of the content typically reviewed by studies in this field of research and ongoing social debate about online privacy and data ownership, whether publicly shared content on social media is of public domain is up for discussion.

## Supporting information

S1 File PRISMA-ScR checklist. (PDF)

S2 File Review protocol. (PDF)

S1 Table Detailed quality appraisal of included studies. (PDF)

## References

[1] Pirkis J, Mok K, Robinson J, Nordentoft M. Media influences on suicidal thoughts and behaviors In: O’Connor RC, Pirkis J, editors. The international handbook of suicide prevention. Hoboken, New Jersey: John Wiley & Sons, Ltd; 2016 p. 743–57.

[2] Niederkrotenthaler T, Braun M, Pirkis J, Till B, Stack S, Sinyor M, et al Association between suicide reporting in the media and suicide: systematic review and meta-analysis. BMJ. 2020;368:m575 10.1136/bmj.m575 32188637 PMC7190013

[3] Ueda M, Mori K, Matsubayashi T, Sawada Y. Tweeting celebrity suicides: Users’ reaction to prominent suicide deaths on Twitter and subsequent increases in actual suicides. Soc Sci Med. 2017;189:158–66. 10.1016/j.socscimed.2017.06.032 28705550

[4] Niederkrotenthaler T, Voracek M, Herberth A, Till B, Strauss M, Etzersdorfer E, et al Role of media reports in completed and prevented suicide: Werther v. Papageno effects. Br J Psychiatry. 2010;197(3):234–43. 10.1192/bjp.bp.109.074633 20807970

[5] Nock MK. Self-injury. Annu Rev Clin Psychol. 2010;6:339–63. 10.1146/annurev.clinpsy.121208.131258 20192787

[6] Jarvi S, Jackson B, Swenson L, Crawford H. The impact of social contagion on non-suicidal self-injury: a review of the literature. Arch Suicide Res. 2013;17(1):1–19. 10.1080/13811118.2013.748404 23387399

[7] Andover MS, Morris BW, Wren A, Bruzzese ME. The co-occurrence of non-suicidal self-injury and attempted suicide among adolescents: distinguishing risk factors and psychosocial correlates. Child Adolesc Psychiatry Ment Health. 2012;6:11 10.1186/1753-2000-6-11 22463065 PMC3379960

[8] Hawton K, Saunders KEA, O’Connor RC. Self-harm and suicide in adolescents. The Lancet. 2012;379(9834):2373–82. 10.1016/S0140-6736(12)60322-5 22726518

[9] Branley DB, Covey J. Is exposure to online content depicting risky behavior related to viewers’ own risky behavior offline? Comput Hum Behav. 2017;75:283–7. 10.1016/j.chb.2017.05.023

[10] Dunlop SM, More E, Romer D. Where do youth learn about suicides on the Internet, and what influence does this have on suicidal ideation?: Influence of the Internet on suicidal ideation. J Child Psychol Psychiatry. 2011;52(10):1073–80. 10.1111/j.1469-7610.2011.02416.x 21658185

[11] Pater J, Mynatt E. Defining digital self-harm. In: Proceedings of the 2017 ACM Conference on Computer Supported Cooperative Work and Social Computing—CSCW ‘17. New York, New York, USA: ACM Press; 2017. p. 1501–13.

[12] Daine K, Hawton K, Singaravelu V, Stewart A, Simkin S, Montgomery P. The power of the web: a systematic review of studies of the influence of the internet on self-harm and suicide in young people. PloS One. 2013;8(10):e77555 10.1371/journal.pone.0077555 24204868 PMC3813687

[13] Dyson MP, Hartling L, Shulhan J, Chisholm A, Milne A, Sundar P, et al A systematic review of social media use to discuss and view deliberate self-harm acts. PLoS One. 2016;11(5):e0155813 10.1371/journal.pone.0155813 27191728 PMC4871432

[14] Krysinska K, Westerlund M, Niederkrotenthaler T, Andriessen K, Carli V, Hadlaczky G, et al A mapping study on the internet and suicide. Crisis. 2017;38(4):217–26. 10.1027/0227-5910/a000444 28228064

[15] Lewis SP, Seko Y. A double-edged sword: a review of benefits and risks of online nonsuicidal self-injury activities. J Clin Psychol. 2016;72(3):249–62. 10.1002/jclp.22242 26613372

[16] Marchant A, Hawton K, Stewart A, Montgomery P, Singaravelu V, Lloyd K, et al A systematic review of the relationship between internet use, self-harm and suicidal behaviour in young people: The good, the bad and the unknown. PloS One. 2017;12(8):e0181722 10.1371/journal.pone.0181722 28813437 PMC5558917

[17] Mok K, Jorm AF, Pirkis J. Suicide-related Internet use: a review. Aust N Z J Psychiatry. 2015;49(8):697–705. 10.1177/0004867415569797 25698810

[18] Instagram. Instagram statistics [Internet]. Info Center. 2020 [cited 2020 Jan 23]. https://instagram-press.com/our-story/

[19] Clement J. Most popular social networks of teenagers in the United States from fall 2012 to spring 2019 [Internet]. Statista. 2019. https://www.statista.com/statistics/250172/social-network-usage-of-us-teens-and-young-adults/

[20] Smith A, Anderson M. Social media use in 2018 [Internet]. Pew Research Center. 2018 [cited 2020 Jan 23]. https://www.pewresearch.org/internet/2018/03/01/social-media-use-in-2018/

[21] The Lancet. Social media, screen time, and young people’s mental health. Lancet. 2019 2 16;393(10172):611–611. 10.1016/S0140-6736(19)30358-7 30782327

[22] Instagram. Introducing type mode stories [Internet]. Instagram Info Center. 2018 [cited 2019 Nov 13]. https://instagram-press.com/blog/2018/02/01/introducing-type-mode-in-stories/

[23] Siegler MG. Instagram launches with the hope of igniting communication through images [Internet]. TechCrunch. 2010. https://techcrunch.com/2010/10/06/instagram-launch/

[24] Instagram. Instagram [Internet]. Instagram.com. 2020. https://www.instagram.com/

[25] Instagram. Exploring photos and videos [Internet]. Help Centre—Using Instagram. 2020 [cited 2019 Nov 20]. https://help.instagram.com/140491076362332/?helpref=hc_fnav&bc[0]=InstagramHelp&bc[1]=UsingInstagram

[26] Crawford A. Instagram ‘helped kill my daughter’ [Internet]. BBC News. 2019. https://www.bbc.com/news/av/uk-46966009/instagram-helped-kill-my-daughter

[27] Fullerton J. Teenage girl kills herself ‘after Instagram poll’ in Malaysia [Internet]. The Guardian. Bangkok; 2019 [cited 2020 Jan 23]. https://www.theguardian.com/world/2019/may/15/teenage-girl-kills-herself-after-instagram-poll-in-malaysia

[28] Savage M. Health secretary tells social media firms to protect children after girl’s death [Internet]. The Guardian. 2019 [cited 2020 Jan 23]. https://www.theguardian.com/politics/2019/jan/26/matt-hancock-facebook-social-media-suicide-self-harm-young-people

[29] Instagram. Changes we’re making to do more to support and protect the most vulnerable people who use instagram [Internet]. Info Center. 2019. https://instagram-press.com/blog/2019/02/07/changes-were-making-to-do-more-to-support-and-protect-the-most-vulnerable-people-who-use-instagram/

[30] Arksey H, O’Malley L. Scoping studies: towards a methodological framework. Int J Soc Res Methodol Theory Pract. 2005;8(1):19–32. 10.1080/1364557032000119616

[31] Tricco AC, Lillie E, Zarin W, O’Brien KK, Colquhoun H, Levac D, et al PRISMA extension for scoping reviews (PRISMA-ScR): checklist and explanation. Ann Intern Med. 2018;169(7):467–73. 10.7326/M18-0850 30178033

[32] Peters MDJ, Godfrey CM, Khalil H, McInerney P, Parker D, Soares CB. Guidance for conducting systematic scoping reviews. Int J Evid Based Healthc. 2015;13(3):141–6. 10.1097/XEB.0000000000000050 26134548

[33] Critical Appraisal Skills Programme. CASP Qualitative Checlist [Internet]. 2018 [cited 2019 Nov 11]. https://casp-uk.net/casp-tools-checklists/

[34] Arendt F. Suicide on Instagram—content analysis of a German suicide-related hashtag. Crisis. 2019;40(1):36–41. 10.1027/0227-5910/a000529 29932019

[35] Arendt F, Scherr S, Romer D. Effects of exposure to self-harm on social media: Evidence from a two-wave panel study among young adults. New Media Soc. 2019;21(11–12):2422–2442. 10.1177/1461444819850106

[36] Scherr S, Arendt F, Frissen T, Oramas MJ. Detecting intentional self-harm on Instagram: development, testing, and validation of an automatic image-recognition algorithm to discover cutting-related posts. Soc Sci Comput Rev. 2019; 10.1177/0894439319836389

[37] Brown RC, Fischer T, Goldwich AD, Keller F, Young R, Plener PL. #cutting: non-suicidal self-injury (NSSI) on Instagram. Psychol Med. 2018;48(2):337–46. 10.1017/S0033291717001751 28705261

[38] Brown RC, Bendig E, Fischer T, Goldwich AD, Baumeister H, Plener PL. Can acute suicidality be predicted by Instagram data? Results from qualitative and quantitative language analyses. Plos One. 2019;14(9):e0220623 10.1371/journal.pone.0220623 31504042 PMC6736249

[39] Carlyle KE, Guidry JPD, Williams K, Tabaac A, Perrin PB. Suicide conversations on Instagram^™^: contagion or caring? J Commun Healthc. 2018;11(1):12–8. 10.1080/17538068.2018.1436500

[40] Moreno MA, Ton A, Selkie E, Evans Y. Secret Society 123: understanding the language of self-harm on instagram. J Adolesc Health. 2016;58(1):78–84. 10.1016/j.jadohealth.2015.09.015 26707231 PMC5322804

[41] Record RA, Straub K, Stump N. #Selfharm on #Instagram: examining user awareness and use of Instagram’s self-harm reporting tool. Health Commun. 2019;1–8. 10.1080/10410236.2019.1598738 30961389

[42] Miguel EM, Chou T, Golik A, Cornacchio D, Sanchez AL, DeSerisy M, et al Examining the scope and patterns of deliberate self-injurious cutting content in popular social media. Depress Anxiety. 2017;34(9):786–93. 10.1002/da.22668 28661053

[43] Shanahan N, Brennan C, House A. Self-harm and social media: thematic analysis of images posted on three social media sites. BMJ Open. 2019;9:e027006 10.1136/bmjopen-2018-027006 30782950 PMC6367987

[44] Liamputtong P. Qualitative research methods. Fourth edition Australia: Oxford University Press; 2013 Chapter 12: Making sense of qualitative data: the analysis process; p.241–63.

[45] Seko Y, Kidd SA, Wiljer D, McKenzie KJ. On the creative edge: exploring motivations for creating non-suicidal self-injury content online. Qual Health Res. 2015 10;25(10):1334–46. 10.1177/1049732315570134 25662942

[46] Goodfellow B, Kõlves K, De Leo D, Silverman M, Berman A, Mann J, et al International study of definitions of English-language terms for suicidal behaviours: protocol of an opinion survey. BMJ Open. 2019 7;9(7):e025770 10.1136/bmjopen-2018-025770 31296506 PMC6624059

[47] Chandler A, King C, Burton C, Platt S. General practitioners’ accounts of patients who have self-harmed: A qualitative, observational study. Crisis. 2016;37(1):42–50. 10.1027/0227-5910/a000325 26572907 PMC4904492

[48] National Institute for Health and Care Excellence [NICE]. Self-harm in over 8s: long-term management (CG133) [Internet]. UK; 2011. https://www.nice.org.uk/guidance/cg133 31891461

[49] Jacob N, Evans R, Scourfield J. The influence of online images on self-harm: a qualitative study of young people aged 16–24. J Adolesc. 2017;60:140–7. 10.1016/j.adolescence.2017.08.001 28881214 PMC5614108

[50] Gerrard Y. Beyond the hashtag: Circumventing content moderation on social media. New Media and Society. 2018;20(12):4492–511. 10.1177/1461444818776611

[51] Moreno MA, Goniu N, Moreno PS, Diekema D. Ethics of social media research: common concerns and practical considerations. Cyberpsychology Behav Soc Netw. 2013;16(9):708–13. 10.1089/cyber.2012.0334 23679571 PMC3942703

