# Supplementary material for: Suicide and self-harm content on Instagram: A systematic scoping review
Source: PLoS One. 2020 Sep 2;15(9):e0238603. doi: 10.1371/journal.pone.0238603 (PMC7467257; doi:10.1371/journal.pone.0238603)
Supplement: S2 File — (PDF) [file pone.0238603.s002.pdf]

Scoping Review of Suicide and Self-harm content on Instagram: Protocol

Research Questions

- *What research has been done (published) about suicide and self-harm on Instagram*
- *How has it been done? What methods have been used?*
- *What are its key findings?*
- *What are the strengths and limitations of the current body of knowledge?*

Search Strategy

Search terms:

(instagram OR "insta gram*") AND (suicid* OR "self harm*" OR selfharm* OR "self injur*" OR selfinjur* OR "self mutil*" OR selfmutil* OR "auto mutil*" OR automutil* OR cut* OR distress* OR disorder* OR anxi* OR depress* OR "psycholog* stress*" OR "psycholog* pain*" OR emotion*).*

** Include mental health related terms potentially associated to self-harm or suicide

Databases to use:

Scopus, Web of Science, Ovid (Psycharticles, Medline, EMBASE, ERIC), EBSCOhost, *ProQuest*.

Papers Selection

Inclusion criteria

- Academic publications (peer-reviewed) journal articles, written in English.

- Papers must explicitly focus on Instagram and suicide or self-harm (that is papers must explicitly state to study and report findings on suicide or self-harm and Instagram).
- Publications published in 2010 and after (when Instagram was launched)

Exclusion criteria

- None peer reviewed journals articles (book chapters, conference proceedings, letters…).
- Articles focused on social media in general, or other social media sites (e.g. Facebook, Twitter…) but not explicitly stating to study Instagram.

Papers selection process

1) Get ALL the listed records for each search at each database.

   **Go through each database independently, searching and extracting all the hits from each database without worrying about duplicates yet.

2) Export all the records from each database to independent folders in Mendeley.
3) Pool all the records together into one folder, and remove duplicates.
4) PRE-SELECTION: From the remaining records, screen them for relevance to the study (apply inclusion/exclusion criteria) based on Title and Abstract.
5) Keep only those records of articles which title & abstract are on topic →1st "A-PRIORI SAMPLE" of studies on the topic.
6) Download those pre-selected articles and read in full.
7) Discard FALSE POSITIVES: Those than when read in full, were found to not really be on topic.
8) From the final selection of full papers, check REFERENCES for possible missed extra relevant papers.
9) FINAL SAMPLE of papers = (1st A Priori Sample – False positives) + Included REFS

Data extraction

Following the 'Data Charting Template'. (see Annex 1).

For every article extract:

- Identification and introductory information (i.e. authors, date and journal of publication, main author affiliation, study aim, and online social media platforms studied)
- Methodological information (i.e. study design, unit of analysis, data collection strategy and sample characteristics, coded/assessed variables, data analysis performed).
- Main reported findings and conclusions.
- Strengths and limitations.
- Quality assessment. (*Adapted version of the CASP qualitative appraisal tools, to fit the different studies). (See Annex 2).

Annex 1: Data charting template

Authors, public.year,	Main author affiliation, (Country)	Title	Declared aims	Study design, and methodology	Age groups	Unit of analysis and Sample size	Language of collected data	Period of data collection	Soc Media Platforms studied	Data analysys	Coding / Variables

– Continued –

	Theoretical frameworks	Main Results	Conclusions	Limitations	Other Comments	Publication	Full Reference

Annex 2: Appraisal checklist

	**Ref Study 1**	**Ref Study2**	**Ref Study3**				
Q1: Aim							
Q2: Methodology							
Q3: Research design							
Q4: Sample							
Q5: Data Collection							
Q6: Analysis rigour							
Q7: Clear findings							
Q8: Acknowledges limitations							
Q9: Ethical considerations							
Q10: Value of research							
Other Comments							

Answers: Yes (Y), No (N), Can’t Tell (CT).

Detailed explanation of the Appraisal Checklist:

We will use a modified version of the Critical Appraisal Skills Program – Qualitative Checklist to undertake the quality appraisal of all included papers.

We chose this quality tool, because its original form is already quite flexible in its assessed criteria, and therefore easy to fit to different heterogeneous papers. Furthermore, in our case, most papers use content analysis, a technique very close epistemologically to qualitative methodologies, despite reporting frequencies and statistical analysis.

Based on the CASP-Qualitative checklist for each included paper we will assess whether it reports:

1) **Aim:** a clear statement of aims.
2) **Methodology:** Originally CASP asks whether qualitative methodology was the appropriate to use. We modified this to assess whether its reported methodology (qualitative or quantitative) was the appropriate for the study aims.
3) **Research design**: Was the research design reported appropriate to address the research aims?
4) **Sample:** Was the recruitment or sampling appropriate for the research aims? Does the paper explain how and why they selected the sample?
5) **Data collection:** Was data collected correctly, in a way that addressed the research issue?
6) **Analysis rigour:** Were data analysis reported sufficiently rigorous? Is there a good description of the analysis process? If data were categorized, is it clear were those categories derived from? Is there sufficient data presented to support the findings?, to what extent contradictory data are taken into account?

7) **Clear findings:** Is there clear statement of findings? Explicit findings, discussed thoroughly. Has the credibility of such findings been discussed? and do the findings relate to the original research question?

8) **Acknowledge limitations:** the researchers acknowledged the study limitations, and openly discussed potential confounders. This item is not originally included in the CASP, but we found it was important to consider it. Ex: Whether they acknowledge issues regarding sampling representativeness, or terminology use….

9) **Ethical considerations:** Have ethical issues been taken into account, and managed accordingly?

10) **Research value.** Was the research valuable? Does the paper discuss the contribution de study makes to existing knowledge? Or considered further ways in which the study knowledge can be used? Do they identify areas were further research is needed?

Every included study in this review will be checked against all these ten items. Whether each of them were YES or NO well addressed, or whether we CAN'T TELL.

** Originally, one item of the Qualitative-CASP weighed whether the relationship between the researcher and the participants had been adequately considered? This aspects were not applicable for any of our piloted papers, where researcher did not play any influential role in data retrieval. Therefore, it is left out from the appraisal assessment.
