# Supplementary material for: Suicide and self-harm content on Instagram: A systematic scoping review
Source: PLoS One. 2020 Sep 2;15(9):e0238603. doi: 10.1371/journal.pone.0238603 (PMC7467257; doi:10.1371/journal.pone.0238603)
Supplement: S1 Table — (PDF) [file pone.0238603.s003.pdf]

Quality appraisal of reviewed studies

Study	Q1: Aim	Q2: Correct methodology	Q3: Research design	Q4: Sample	Q5: Data collection	Q6: Analysis rigour	Q7: Clear findings	Q8: Acknowledges limitations	Q9: Ethical considerations	Q10: Value of research	Score (criteria well met)
Moreno 2016	Y	Y	Y	Y	Y	Y	Y	Y	Y	Y	10/10
Miguel 2017	Y	Y	Y	Y	Y	Y	Y	Y	Y	Y	10/10
Brown 2018	Y	Y	Y	Y	Y	Y	Y	Y	Y	Y	10/10
Carlyle 2018	Y	Y	Y	Y	Y	Y	Y	Y	N	Y	9/10
Shanahan 2019	Y	Y	Y	Y	Y	Y	Y	Y	Y	Y	10/10
Scherr 2019	Y	Y	Y	Y	Y	Y	Y	Y	Y	Y	10/10
Arendt 2019	Y	Y	Y	Y	Y	Y	Y	Y	Y	Y	10/10
Record 2019	Y	Y	Y	Y	Y	Y	Y	Y	Y	Y	10/10
Arendt, Scherr 2019	Y	Y	Y	Y	Y	Y	Y	Y	Y	Y	10/10
Brown 2019	Y	Y	Y	Y	Y	Y	Y	Y	Y	Y	10/10

Possible answers: YES (Y), NO (N) or CAN'T TELL (CT)
